# Annotations

**Published:** 1890-06-21

**Authors:** 


### Annotations.

**Mrs. Gladstone's Appeal.**

Mrs. Gladstone is in a state of considerable anxiety about a very useful charity of the minor sort. It is an institution of a kind greatly needed in large towns; and it ought not, on any account, to be allowed to be crowded out by the too urgent competition of other charities. The institution is the Newport Market Industrial School and Refuge. The fact that it is twenty-five years old is some evidence in its favour; and the other two facts, that it has done a great deal of useful work, and that it has done it very economically, are together convincing proofs that it has earned its right to survive and be supported. Philanthropic readers will do well to consider both the kind and quantity of a single year's work, as given by Mrs. Gladstone in a recent appeal to the public through the newspapers. The work is that of last year, and is sufficiently various. Among other things done, a hundred and twenty men who were out of work were placed in situations, and twenty more were specially assisted in different ways. A good many women presented themselves at the refuge during the year, and of these no fewer than eighty-four were rescued from a bad life. Of other women who applied, a hundred and sixteen were provided with employment, and seventy-one were temporarily helped. The school at the refuge educated during the year eighty-five boys. Of those twenty-five passed out, and their places were more than filled by thirty-eight new admissions. Patriotic readers will be glad to learn that the greater number of the boys who passed out entered military bands, the school having for many years enjoyed a high reputation as a nursery for bandsmen. Now it is evident that all this is exceedingly good work. Moreover, institutions that do this kind of public service are not half so numerous as they ought to be. But Mrs. Gladstone informs us that "all this good work is in imminent danger of collapse." Why is this? It is because many of the original friends of the Refuge have died, and it has been so difficult as to be almost impossible to supply their places. That ought not to be, in a country where there are so many people who can afford to give a little more than they do at present. Mrs. Gladstone's sponsorship may be taken as a sufficient guarantee both of the excellence of the work done and the economy of the management. We cannot but think that her simple and touching appeal will find a responsive echo in many thoughtful and kindly hearts. The real value of the services rendered can never be known except by the men who are saved from destitution and the women who are delivered from a life worse than death. The gratitude of these will be cheaply earned by such modest help as Mrs. Gladstone asks.

**"Brain-fag" in Women.**

All generous and enthusiastic women, and more particularly those who are young, will do well to lay to heart the story of the melancholy death of Miss Mary L. G. McGaw, the daughter of the Rev. J. T. McGaw, of Manchester. Miss McGaw was well-known in Sale and Manchester as a zealous and successful worker in connection with the Prison Gate Mission and other agencies for raising the fallen. She worked incessantly, and much beyond her strength. The result was that a condition of extreme brain exhaustion accompanied by melancholia was brought about. By the advice of an eminent London physician she was placed under the care of an experienced nurse at Brixton Hill a few weeks ago. Her health improved so much that she was permitted by the nurse to go out alone. On the 7th inst. she went to leave an order at a shop, but never returned. Her body was found in the Thames a few days afterwards. The devotion of young people, both men and women, to such objects as the restoration of the fallen and wicked to honest and wholesome ways of living, is beyond all praise. To say a word about it of any kind, except commendation, is difficult. But religion as well as science demands that such work be done in measure and with reasonable obedience to the laws of healthy life. Youthful zeal is often very effective in good works, but it is also very consuming. If the work could not possibly be done except at the cost of noble young lives, we might be willing even to go so far as to consent to their premature sacrifice. But when zeal is coupled with ripe experience the power of effective work is increased tenfold. Young people cannot be expected to know this of themselves, or even to believe it very readily when they are told. But their elders, and particularly their parents and religious teachers, may reasonably be expected to know it. Too often religious teachers strive to stimulate and excite young people, until their zeal and enthusiasm become dangerous to reason and health. No doubt many young women are frivolous, and require strong religious excitation in order to be roused to even the most commonplace spiritual levels. But discrimination must be used. The tender consciences and highly-wrought minds of the nobler class of young women are not fit objects for whip and spur. Not only must parents and religious teachers exercise more directing control over generous-minded young women, but girls themselves must try to learn that, as the world was not created in a day, so neither can it be converted in a day. The patience of Almighty God, who permits the wheat and tares to grow together until the harvest, must be regarded as a permanent object lesson. Three or four years of zealous and enthusiastic work may undoubtedly accomplish much, even if experience be limited; but thirty or forty years of the same enthusiasm with constantly growing experience will effect ten thousand times more. The world can ill spare such generous and noble workers as Mary McGaw. The first symptoms of brain exhaustion ought always to be regarded as the providential striking of the hour when work should temporarily cease; and rest should then be sought until the energies are completely restored.

**Jews and Athletics.**

In the days of Samson and in those of King David it is probable that there were many Jews who could give a good account of themselves in a hand-to-hand conflict, either with man or savage beast. But physical robustness, as distinguished from agility and staying power, has not lately been the distinguishing characteristic of the Jew, at any rate in modern London. What is one of his marked features is an intense practicality, with the power of seeing and laying hold of all kinds of circumstances that make for his advantage. Last week the Jewish School at Stepney, of which Lord Rothschild is a patron, and Mr. Marcus Adler, M.A., a son of the late chief Rabbi, is the president, celebrated its annual prize distribution in a fashion as novel as it was interesting. The function was carried out in Mr. Charrington's great hall, at Mile End, and there were nearly three thousand visitors present, all Jews, except, perhaps, the vicar of the parish and the present writer. The principal part of the programme consisted of an elaborate display of gymnastic exercises, carried out by girls and boys alternately. The girls were prettily dressed in white loose robes with white turbans and sashes of pale blue. The boys were also attired in the ordinary "whites" of the gymnasium. The floor of the vast hall gave ample space, and the various dumb-bell, crossbar, pole, and other exercises were gone through with the utmost spirit and energy, and with elaborate precision. Burst after burst of applause greeted the youthful performers as they displayed some more than ordinarily graceful evolutions, or performed feats requiring more than usual agility and strength. The president, Mr. Adler, expressed the conviction that such physical training was invaluable both for boys and girls. He referred also to the technical instruction given to the elder pupils in carpentery, smith's work, cooking, needle-work, and the like. Hitherto, he said, the Jews of the East-end had principally entered the trades of shoemaking, tailoring, cigarette folding, and so on, trades which did not require great physical strength. But their choice of those occupations had been decidedly injurious, and most of all to the Jews themselves. He advised the boys to devote as much time as possible both to gymnastics and to technical work, in order that when they left school they might be fitted for the healthier and the better-paid trades. This new departure is due chiefly to the discerning intelligence of Mr. W. Ashe Payne, head master of the school, and has been rendered possible by the facilities furnished for technical instruction and gymnastics at the People's Palace. It is more than probable that all the public elementary schools of the East-end will in course of time follow the example of the Jewish School and become associated with Sir Edmund Currie and the People's Palace for technical instruction and physical training. When it is added that according to careful actuarial calculations this East-end Jewish school of 800 very poor children has earned during the past year a higher all-round Government grant than any other public elementary school in the metropolis or even in England, the value of physical training for poor children is demonstrated beyond a doubt.

